# Sudden Vasopressin Withdrawal Causing Transient Central Diabetes Insipidus: A Case Report

**DOI:** 10.7759/cureus.24966

**Published:** 2022-05-13

**Authors:** Ramakanth Pata, Nway Nway, Ilana K Logvinsky, Innocent Lutaya, Tutul Chowdhury

**Affiliations:** 1 Pulmonary and Critical Care Medicine, One Brooklyn Health, New York, USA; 2 Pulmonary and Critical Care Medicine, University of Cincinnati Medical Center, Cincinatti, USA; 3 Internal Medicine, Interfaith Medical Center, New York, USA; 4 Medicine, American University of Antigua, New York, USA; 5 Internal Medicine, One Brooklyn Health System, New York, USA

**Keywords:** adh, central diabetes insipidus, diabetic insipidus, antidiuretic hormone, vasopressin

## Abstract

Vasopressin is a peptide hormone produced by the hypothalamus and stored in the posterior pituitary. It is secreted in response to hypotension and hyperosmolarity. Vasopressin and its analogs have been widely used in vasodilatory shocks such as septic shock and cardiogenic shock. The sudden withdrawal of vasopressin after its prolonged use can lead to polyuria and rising sodium levels, which is concerning for the diagnosis of diabetic insipidus (DI); likely central rather than nephrogenic in origin. We present a case of diabetic insipidus following the sudden discontinuation of a prolonged vasopressin infusion for septic shock, which responded to tapering doses of desmopressin.

## Introduction

Vasopressin is an analog of antidiuretic hormone (ADH) that is widely used in the intensive care unit (ICU) under several clinical scenarios, including, but not limited to, advanced vasodilatory septic shock, digestive bleeding due to esophageal and gastric varices [1]. Adults diagnosed with severe sepsis have relatively low levels of vasopressin, requiring the use of exogenous vasopressin for hemodynamic support on escalating doses of norepinephrine. Vasopressin use is further supported by the growing evidence of its safety and efficacy in septic shock [1-4]. In addition, vasopressin analogs are also a mode of treatment for hypothalamic diabetes insipidus (DI), which is characterized by a hypotonic urinary output of more than 3 liters a day. This is because of its anti-diuretic properties [5]. We describe a patient requiring prolonged vasopressin infusion for approximately seven days, followed by a sudden withdrawal, resulting in the development of clinical and laboratory manifestations suggestive of diabetes insipidus. This acquired diabetes insipidus responded to tapering doses of desmopressin. Very few cases have been reported in the literature describing this rare outcome of sudden Vasopressin withdrawal.

## Case presentation

A 52-year-old man with no known past medical history was transferred to our ICU for the initiation of venovenous extracorporeal membrane oxygenation (VVECMO). He was initially admitted to a regional hospital four days earlier for COVID-19-related acute respiratory distress syndrome (ARDS). He was managed at the regional hospital with empiric antibiotics, dexamethasone (DEXA-ARDS protocol), remdesivir, and lung-protective ventilation requiring prone positioning and paralysis. He was then transferred to our hospital for further management with ECMO as rescue therapy. As there were no contraindications, after satisfying EOLIA criteria, he was immediately cannulated and started on an ECMO with a VV configuration. Mechanical ventilation was continued on a pressure-controlled ventilator with a minimal setting (FiO_2_ 40%, positive end-expiratory pressure [PEEP] of 8 cm H_2_O, and pressure control of 20 cm H_2_O). Any recruitment strategy was unsuccessful, and the settings were based on lung-protective ventilation. Lung recovery was monitored via tidal volume and inspiratory flow. Standard ECMO labs and monitoring were done as per the institutional policy. Blood flow and sweep gas flow were adjusted according to blood gas analysis. Due to prolonged steroid use (for unresolving ARDS), pneumocystis jiroveci pneumonia prophylaxis, initially with Bactrim, and then switched to dapsone. His hospital course was further complicated by a right lower lobe segmental pulmonary embolism treated with heparin infusion.

Bronchoscopy with bronchoalveolar (BAL) lavage was done on day 4 as the opacities on the chest X-ray worsened and new-onset hypotension required vasopressors. Broad-spectrum antibiotics were initiated that included vancomycin and cefepime. Vasopressors, including high-dose norepinephrine and vasopressin, were added at a fixed dose of 0.03 u/hour to the norepinephrine due to worsening pneumonia. Blood cultures and BAL cultures were negative.

An echocardiogram revealed the normal systolic function of the left ventricle with an EF of 60-65%. The right ventricle was dilated with a tricuspid annular plane systolic excursion of 1.5 cm and moderate tricuspid regurgitation. The right ventricular systolic pressure was around 60 mm Hg.

From day 8 (four days after starting vasopressin), sodium levels gradually started to decline, reaching a nadir of 125-127 mmol/L. The patient clinically appeared fluid overloaded and both the fluid overload and hyponatremia were managed with furosemide infusion for 24 hours. As it was a gradual decline in serum sodium (>48 hours), the urgent correction was not deemed necessary and hypertonic saline was not administered. Cortisol and thyroid function tests returned normal. On day 11 (seven days of vasopressin), the patient was able to be weaned off of norepinephrine and vasopressin. On day 12, serum sodium levels rapidly rose from 125 to 143 meq/L with a urine output of more than 6 L, accompanied by low urine osmolality <250 mosm/L. Because the rise in serum sodium was acute (<24 hours), hypotonic fluids were administered to match the urine output. This required the administration of almost 500 cc of intravenous fluid per hour. To minimize further polyuria, desmopressin 2 mcg IV was given. The administered desmopressin increased the urine osmolarity (672 mosm/L), decreased urine output significantly, and serum sodium returned to 130. A presumptive diagnosis of diabetes insipidus was made based on the response to the administered desmopressin. The response to desmopressin was consistent with central diabetes insipidus (CDI). A CT scan of the head was obtained to rule out a pituitary lesion, which revealed no acute intracranial abnormality, except for mild diffuse cerebral volume loss. An MRI brain was not obtained as the patient was on ECMO and it was logistically deemed risky.

From 12 hours after the administration of desmopressin, the urine output and serum sodium started rising again with a drop in urine osmolality. This required a second dose of desmopressin 2 mcg IV that temporarily minimized the fluctuations in serum sodium. On day 13, desmopressin 2 mcg Q12 IV was administered. On day 14, desmopressin 0.2 mg Q12 was administered via NG tube and urine osmolality, urine output, and serum sodium were monitored every four hours. On day 16, the desmopressin dose was decreased to 0.1 mg Q12. On day 18, the dose could be further decreased to 0.05 mg Q12. Desmopressin was discontinued on day 19 after the administration of a single dose. No specific weaning protocol was followed, but the dose was adjusted by monitoring urine osmolarity, urine output, and serum sodium with the premeditated intention of desmopressin weaning. After the discontinuation of desmopressin, the serum sodium plateaued with normalization of urine output and urine osmolarity.

**Figure 1:**
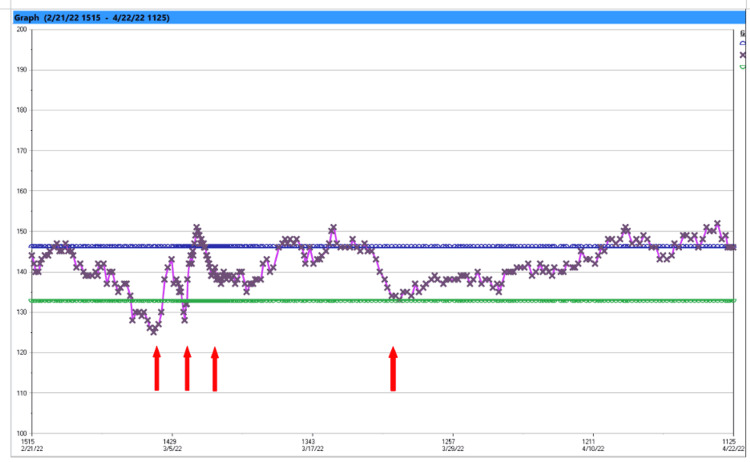
Graph displaying serum sodium concentration. First arrow: first dose of desmopressin 2 mcg IV administered. Second arrow: second dose of desmopressin 2 mcg IV. Third arrow: oral desmopressin 0.2 mg NGT Q12 initiated with an intention of taper. Fourth arrow: oral desmopressin discontinued.

**Figure 2:**
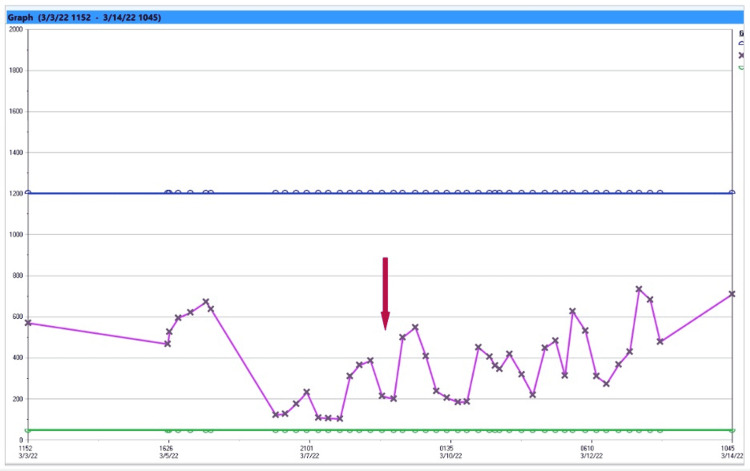
Trend of urine osmolarity. Fluctuations in the urine osmolality represent the response of urine osmolality increasing to a dose of desmopressin and decrease in osmolality as the effect of desmopressin dose weaned off (see arrow). It should be noted that urine osmolarity did not reflect serum sodium as the combination of both desmopressin and fluid therapy was considered to prioritize serum sodium to remain as stable as possible. Intravenous fluid dose was calculated based on hourly urine output.

**Figure 3:**
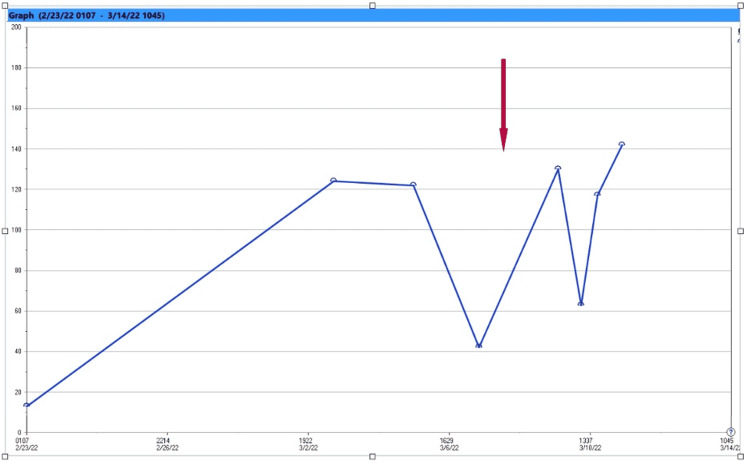
Trend in urine sodium. The arrow represents the fluctuation of urine sodium in response to desmopressin.

**Figure 4:**
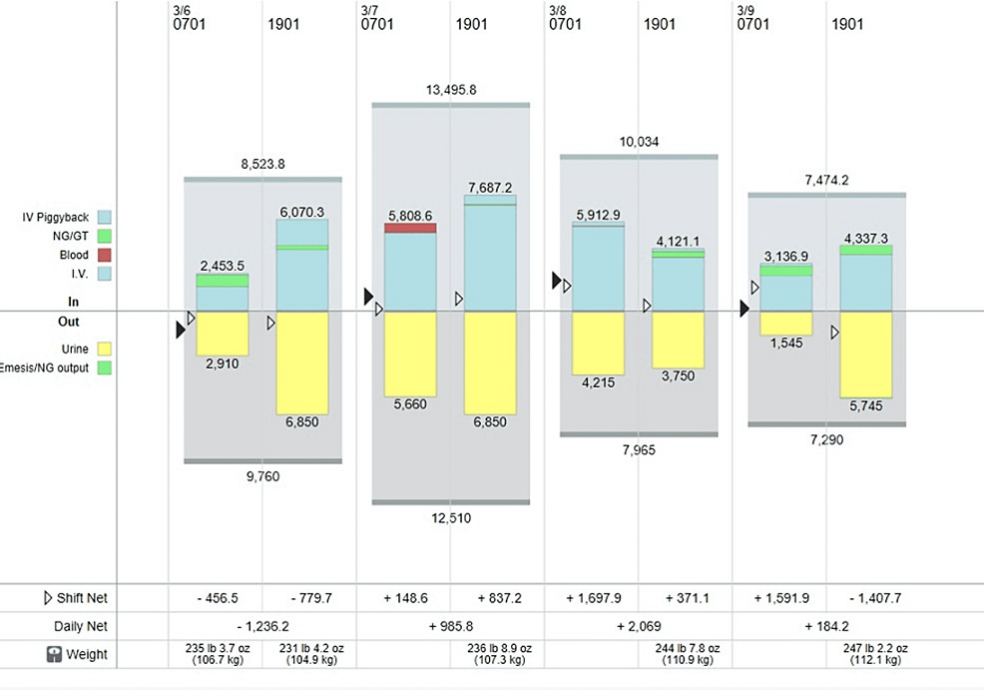
Daily intake and output during vasopressin withdrawal and administration of desmopressin. As it can be seen in the graph on March 7, 2022, the intravenous fluid therapy was given based on an hourly urine output almost reaching 7.5 liters/day.

**Figure 5:**
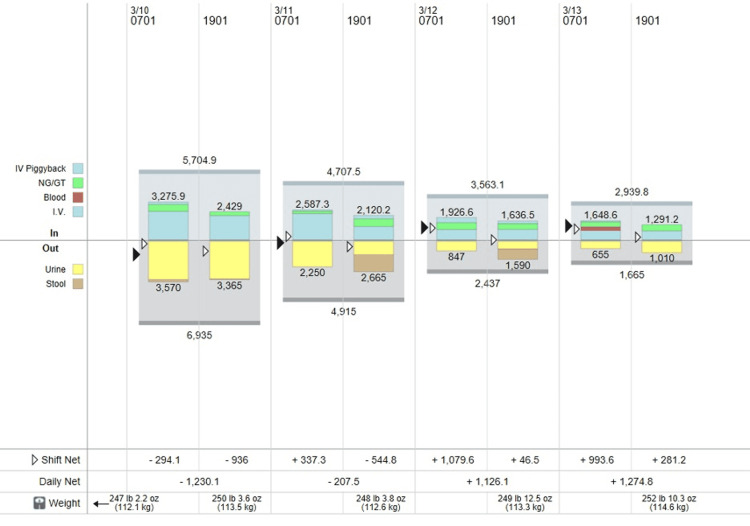
Daily intake and output during administration of desmopressin. The urine output gradually decreased from 6.9 L/day to 1.6 L/day when the desmopressin was discontinued.

The trends in urine osmolality, urine output, and serum sodium are presented in Figures 1-5.

## Discussion

Vasopressin (ADH) is a neuropeptide synthesized in the hypothalamus and released by the posterior pituitary in response to thirst, hyperosmolality, and hypotension. It works on the aquaporin channels in the kidney tubules and preferentially absorbs water without sodium, thus regulating close water balance. It also exhibits vasoconstrictive properties. Deficiency of vasopressin or resistance to the effect of vasopressin results in polyuria, nocturia, and polydipsia. This results in the preferential excretion of large quantities of water in the urine without sodium or urea, making it "insipid." This has been referred to as diabetes insipidus. A triad of hypernatremia, increased urine output, and decreased urine osmolality (<250 mosm) is pathognomonic of diabetes insipidus. Untreated DI patients' blood sodium levels are frequently in the high normal range, which stimulates thirst to replenish urinary water losses. When thirst is inhibited or cannot be expressed, moderate to severe hypernatremia can occur.

Disorders affecting the hypothalamus osmoreceptors, supraoptic or paraventricular nuclei, or the superior section of the supraoptic hypophyseal tract can all lead to a lack of ADH [6]. In approximately 30% to 50% of cases of central diabetes insipidus, hormone-secreting cells in the hypothalamus are destroyed. In some cases, an autoimmune process is suspected to be involved, causing inflammation in the pituitary stalk and posterior pituitary, which resolves upon the destruction of the targeted neurons [7,8].

A deficiency of ADH or impaired release of ADH is referred to as central diabetes insipidus and typically responds to the administration of desmopressin. In the case of CDI, the cause may be neurosurgery (usually transsphenoidal) or trauma to the posterior pituitary or hypothalamus. As a result of transsphenoidal removal of an adenoma confined to the sella, the incidence of CDI can range from 10-20% to 60-80% with larger tumors. The hypothalamic-pituitary region may be affected by primary or secondary tumors in the brain (most often due to cancers of the lung, leukemia, or lymphoma) [9,10]. People with Langerhans cell histiocytosis (also known as histiocytosis X and eosinophilic granuloma), who suffer from hypothalamic-pituitary diseases, are at high risk for CDI [11].

Resistance to the action of the antidiuretic hormone causes nephrogenic diabetes insipidus, which is defined as a reduction in urine concentrating capacity. Nephrogenic diabetes insipidus does not respond to the administration of desmopressin. Some genetic causes are due to a deficiency in the aquaporin-2 gene, which encodes the ADH-sensitive water channels in the collecting tubule cells [12]. Drugs, such as lithium, which cause the aquaporin-2 water channel to malfunction [13]. Hypercalcemia over 11 mg/dL (2.75 mmol/L) or severe hypokalemia can interfere with ADH's ability to increase collecting tubule water permeability [6,14].

Our patient did not have any significant past medical history of autoimmune diseases or neurosurgery. He received seven days of continuous infusion of vasopressin at a fixed dose of 0.03 u/hr as recommended by the surviving sepsis guidelines for hemodynamic support as an adjunct to norepinephrine. Shortly after the discontinuation of vasopressin, serum sodium rose up with a drop in urine osmolality and increasing urine output. This appropriately responded to the administration of desmopressin. Ferenchick et al. have published a retrospective study of potential diabetes insipidus after discontinuation of vasopressin. It has been proposed that the mechanism of diabetes insipidus after discontinuation of vasopressin infusion may involve transient downregulation of V2 receptors induced by exposure to supraphysiological doses of vasopressin [15]. However, this should cause a nephrogenic diabetes insipidus as a downregulated receptor would mean vasopressin resistance. Our patient appropriately responded to exogenous administration of desmopressin, suggesting probable feedback inhibition on the release of ADH due to vasopressin infusion. Nevertheless, it is important to be aware of these manifestations as fluctuations in serum sodium are associated with morbidity, including neurological dysfunction and atrial fibrillation [16]. Furthermore, increased urine output may affect the estimation of GFR and may result in suboptimal antimicrobial dosing [17].

This unique manifestation of diabetes insipidus after the withdrawal of vasopressin infusion should be considered as a transient or an acquired form to prevent mislabeling this potentially transient phenomenon as central diabetes insipidus. Our patient responded very well to the gradual weaning off of desmopressin, and we recommend initiating desmopressin with the intention of weaning while monitoring urine output and urine osmolality.

## Conclusions

The patient in this study was on prolonged vasopressin use, which inadvertently shut off the ADH production in the hypothalamus, thus disrupting the hypothalamic-pituitary axis and transport of ADH to the kidneys. Abrupt withdrawal of vasopressin, as in this case, resulted in an acquired form of central diabetes insipidus where the body did not have sufficient time to generate ADH. Administering desmopressin, which has a potent antidiuretic but not a vasopressor activity, is used to decrease urine output until ADH can be formed in the hypothalamus and can regain its release kinetics.

The case we presented sheds light on the development of a transient form of central diabetic insipidus following the withdrawal of vasopressin infusion. This case demonstrates the importance of proactively monitoring urine output, sodium levels, and urine osmolarity in patients undergoing prolonged vasopressin infusion to prevent CDI upon sudden discontinuation of this routine care.

## References

[1] Xiao X, Zhu Y, Zhen D, Chen XM, Yue W, Liu L, Li T (2015). Beneficial and side effects of arginine vasopressin and terlipressin for septic shock. J Surg Res.

[2] Xiao X, Zhang J, Wang Y (2016). Effects of terlipressin on patients with sepsis via improving tissue blood flow. J Surg Res.

[3] Kampmeier TG, Rehberg S, Westphal M, Lange M (2010). Vasopressin in sepsis and septic shock. Minerva Anestesiol.

[4] Morelli A, Ertmer C, Pietropaoli P, Westphal M (2009). Terlipressin: a promising vasoactive agent in hemodynamic support of septic shock. Expert Opin Pharmacother.

[5] Garrahy A, Thompson CJ (2020). Management of central diabetes insipidus. Best Pract Res Clin Endocrinol Metab.

[6] Rose BD, Post TW (2001). Clinical Physiology of Acid-Base and Electrolyte Disorders.

[7] Pivonello R, De Bellis A, Faggiano A (2003). Central diabetes insipidus and autoimmunity: relationship between the occurrence of antibodies to arginine vasopressin-secreting cells and clinical, immunological, and radiological features in a large cohort of patients with central diabetes insipidus of known and unknown etiology. J Clin Endocrinol Metab.

[8] Maghnie M, Cosi G, Genovese E (2000). Central diabetes insipidus in children and young adults. N Engl J Med.

[9] Ghirardello S, Hopper N, Albanese A, Maghnie M (2006). Diabetes insipidus in craniopharyngioma: postoperative management of water and electrolyte disorders. J Pediatr Endocrinol Metab.

[10] Kimmel DW, O'Neill BP (1983). Systemic cancer presenting as diabetes insipidus. Clinical and radiographic features of 11 patients with a review of metastatic-induced diabetes insipidus. Cancer.

[11] Grois N, Fahrner B, Arceci RJ (2010). Central nervous system disease in Langerhans cell histiocytosis. J Pediatr.

[12] Fujiwara TM, Bichet DG (2005). Molecular biology of hereditary diabetes insipidus. J Am Soc Nephrol.

[13] Grünfeld JP, Rossier BC (2009). Lithium nephrotoxicity revisited. Nat Rev Nephrol.

[14] Peterson LN, McKay AJ, Borzecki JS (1993). Endogenous prostaglandin E2 mediates inhibition of rat thick ascending limb Cl reabsorption in chronic hypercalcemia. J Clin Invest.

[15] Ferenchick H, Cemalovic N, Ferguson N, Dicpinigaitis PV (2019). Diabetes insipidus after discontinuation of vasopressin infusion for treatment of shock. Crit Care Med.

[16] Timilsina S, Pata R, Timilsina S, Cherala S, Kafle P (2019). Correction of hypernatremia due to pure dehydration could be a potential risk factor for transient atrial fibrillation. Cureus.

[17] Pata RK, Bastola C, Nway N, Patel MJ, Adhikari S (2021). Augmented renal clearance in a case of sepsis leading to vancomycin failure despite increasing dose as per the estimated glomerular filtration rate. Cureus.

